# Deployment of small cells and a transport infrastructure concurrently for next-generation mobile access networks

**DOI:** 10.1371/journal.pone.0207330

**Published:** 2018-11-26

**Authors:** Welton Araujo, Rafael Fogarolli, Marcos Seruffo, Diego Cardoso

**Affiliations:** Operational Research Laboratory, Federal University of Pará, Belém, Pará, Brazil; City University of Hong Kong, HONG KONG

## Abstract

The exponential growth of mobile traffic means that operators must upgrade their mobile networks to provide higher capacity to final users. A promising alternative is to deploy heterogeneous networks (HetNets) that combine macro Base Stations (BSs) and SmallCells (SCs), although this increases the complexity and cost of the transport (SCs to Fiber Access Point–FAP). Most of the planning strategies outlined in the literature are aimed at reducing the number of SCs and ignore the impact that the transport segment might have on the total cost of network deployment. In this paper, heuristics are used for the joint planning of radio (i.e., SCs) and transport resources (i.e., point-to-point fiber links). These were compared and examined to determine the advantages and disadvantages of each approach, and in some cases, this led to a 50% reduction in total costs, while still creating a non-scalable network.

## Introduction

Some industrial and academic specialists predict that Global IP traffic will increase nearly threefold over the next 5 years, and will have increased 127-fold in the period from 2005 to 2021. Overall, IP traffic will grow at a Compound Annual Growth Rate (CAGR) of 24 percent from 2016 to 2021 [1][2]. Allied to this growth, other new types of internet connection are emerging, that have led to new networks like Vehicular Networks (VN) [3] and Wireless Sensor Networks (WSN) [4–8]; it is predicted that these will be combined with others, forming a new paradigm called Internet of Things (IoT) [9].

With the development of information network, the popularity of IoT is an irreversible trend and generating new demands that must be assured. Examples are IoT demands [10–12], impacts, and implications on sensors technologies [13–15], big data management [16][17], and future Internet design for various IoT use cases, such as smart cities, smart environments [18–22], smart homes, etc.

These new technologies demand even more capacity, not only in terms of throughput but also in latency, and this will require a considerable investment in the mobile network infrastructure. The Fifth-Generation (5G) of mobile networks encompasses all the requirements of IoT and has the capacity to interconnect all existing and emerging technologies.

However, improvements in the physical layer alone cannot support such a high data rate [2][23], and there are many other means of improving the data rate of mobile users in HetNets, e.g., bandwidth aggregation [24], multipath transport [25][26] and in particular, the extreme densification of heterogeneous SCs.

According to the Small Cell Forum [27], “Small Cell” is an umbrella term for operator-controlled, low-powered radio access nodes, including those that operate in licensed spectrum and unlicensed carrier-grade Wi-Fi. The coverage of SCs generally ranges from ten to several hundred meters. The types of SCs include femtocells, picocells and microcells–and these tend to increase in size from femtocells (the smallest) to microcells (the largest).

The deployment of SC networks raises several challenges; authors in [28] investigated their criticalness and according to the findings of a survey for public SC deployments, the backhaul network, electric power, and SC placement strategies, were considered to be the most serious issues. Backhaul can be regarded as the most crucial factor that affects SC deployments, followed by questions related to scenarios in a coexisting macrocell environment [29]. Thus, Radio Network Planning (RNP) is essential to enable operators to deploy wireless cellular networks in a cost-effective manner, and include both radio and transport networks.

There are many possible means of improving the transport network, including the use of copper wire, microwave, and fiber optics. However, most of the current studies related to the base station deployment have focused on radio deployment scenarios without taking account of the transport network. Even when this factor is included, they are concerned with scenarios in which high-speed, fiber-enabled and wired backhaul sites, are available everywhere [30–32], a situation that is considered to be unrealistic by [33]. The cost incurred by deploying a high-capacity backbone network for SCs can be quite high. The authors in [29] studied the optimal placement of SCs and examined scenarios with limited fiber access nodes; however, these fail to take into account the user’s minimum capacity requirements or the problem of radio interference.

Thus, while previous studies have examined serious deployment problems, to the best of our knowledge, none of them has addressed the question of joint deployment of SCs with fiber access for backhaul nodes, which include minimum capacity requirements, interference mitigation techniques and user distribution. In light of this, this paper seeks to make the following contributions: i) a new set of heuristics for SC deployment that includes factors such as minimum Quality of Service (QoS), coverage and SC interference; ii) a discussion of the applicability of the proposed heuristics and others from the literature, based on the results of simulations.

The paper is structured as follows: first, there is an overview of the problem of deployment in SC networks including transport features, which involves carrying out a review of related works on this subject. Following this, there is an examination of the heuristics, together with the network elements concerned. Finally, the results are analyzed and the conclusions are summarized, along with some suggestions for future works in this area.

## Related works

The deployment of HetNets comprising of Small Cells with a fiber-based transport system is expected to be a very attractive means of providing coverage and capacity in densely populated areas. A fiber-based backhaul solution offers the high capacity needed to meet this requirement, but it is costly [2] and time-consuming to deploy, when not readily available. Hence, when deploying the infrastructure of next-generation cellular systems, backhaul links should be included in combination with SCs to reduce network costs and optimize performance.

There have been extensive studies of RNP in the literature because of its importance. For example, Guo et al. [34] established a theoretical framework to maximize the spectral efficiency of the network and avoid interference caused by SC deployment. Cheng et al. [35] and Shimodaira et al. [36] adopted the throughput of a system as a performance metric to find optimal locations for placing small static cells. Coletti et al. [37][38] devised outage deployment mechanisms in realistic metropolitan scenarios.

In [39], a promising strategy was employed to offload a significant amount of data from a macro BS through an SC placement service. This approach was adopted as a means of dimensioning Long Term Evolution (LTE) cellular networks so that the number of BSs required to cover an area of interest could be determined. It had to take into account factors such as user density, service subscriptions, resource allocation, and interference mitigation. In [40], the approach was extended to the use of simulated annealing for HetNets.

In [41] a greedy micro BS deployment strategy was employed over the existing macro cellular network with the aim of maximizing the energy efficiency of the network while meeting the growing demand for capacity. In [42], Xu et al. propose a Q-learning based network selection algorithm for a heterogeneous wireless network scenario and found a solution that can achieve a good performance in terms of blocking probability. In [43], Helou et al. recommend a network-assisted approach for radio (BS) selection, with the aim of improving network performance and user experience.

Network-centric and user-centric strategies are set out in [44], where the authors examine the resource allocation problem by determining the number of resources that must be assigned to the users by each BS. Both strategies involve conducting an analysis of a multihoming approach.

Previous recommendations have made significant contributions to the deployment of SCs. Although, in the author´s opinion, this challenge has not been fully investigated. For this reason, this study supplements previous research studies by offering new strategies (considering multiple features like interference, Signal-to-Interference-Plus-Noise Ratio, coverage and minimum QoS) and comparing them with others in the literature.

## Basic features of SCs and transport deployment strategies

SC deployment traditionally calculates coverage on the basis of traffic density. This traffic is difficult to characterize, especially in view of its dynamic nature and the shifting trends in usage patterns and social mobility. Nonetheless, according to [45], a great deal of traffic information can be inferred and forecasts made on the basis of the following: i) demographics: the distribution of the residential and business community on the basis of demographic data; ii) the traffic system: vehicular data based on public transport and the movement patterns of private vehicles; iii) fixed line data plans: based on a correlation with fixed line phone call records, (most mobile data traffic occurs indoors).

Machine learning algorithms are described as learning a target function that best maps input variables to an output variable and are applied in several areas like routing protocols for different types of users and networks [46–50], TCP/IP protocols optimization [51][52], energy efficiency [53], data classification [54], and etc. In the case of SCs deployment, when there is a set of possible cell-site locations, iterative techniques are usually used to scan the optimal locations. Optimization methods such as integer programming, simulated annealing, and genetic programming algorithms, can be employed to search for optimal solutions.

The mobile SC deployment problem is an NP-hard problem and this can be proved by a reduction from the SCs facility location problem. The proof is not included here owing to a lack of space but was explained in [55], where the authors formulated an optimized SC deployment problem with the aim of maximizing the service time provided by small mobile cells for all users, while taking account of a finite number of small mobile cells and inter-cell/cross-cell interference. Moreover, in the same study, it was proved that this is a NP-hard problem. The paper in [56] studied SC deployment in existing HetNets and stated that this is an NP-hard problem too.

A good solution was found in [33], where a commercially available CPLEX linear programming solver was used to establish an optimization framework. However, as pointed out in the paper, the computation time was very significant and depended on the scale of the dataset. If there is a need to plan a SC network for a large region, a heuristic approach may be required to achieve a satisfactory result within a reasonable period.

The authors in [45] state that there is a temptation to deploy SC without articulated radio planning and rely on signal processing techniques to improve the performance. The danger of adopting this approach is that it is hampered by a lack of effective interference mitigation techniques and also involves a huge increase in the network deployment costs. For these reasons, this paper both uses and compares heuristics for SC and backhaul deployment, by including real-world factors such as the following: the existence of fiber resources that are sparsely located, interference, costs, coverage and QoS.

## Network Parameters

The downlink Signal-to-Interference-Plus-Noise Ratio (SINR) over a given subcarrier *n* assigned to user *k*, can be expressed as follows:

$$SINR_{k}=\frac{P_{k,b\left(k\right)}}{\sigma^{2}+I_{k}}.$$ (1)

in which $P_{k,b(k)}$ is the received power on subcarrier *n* assigned to user *k* by its serving BS *b(k)*; $\sigma^{2}$ is the thermal noise power; and $I_{k}$ is the inter-cell interference from neighboring SCs. It was assumed that all the SCs are transmitting with maximum power *PS*. The received power at user *k* from *b(k)* can be calculated by means of (2), which relates the received power to a node and is the result of the transmitted power and the fading of the signal calculated by the Stanford University Interim (SUI) model [57] This can be expressed as:

$$P_{k,b\left(k\right)}=\frac{10^{\frac{Pot_{b\left(k\right)}-L_{SUI}}{10}}}{1000}.$$ (2)

The value of $L_{SUI}$ is calculated by the three equations shown below:

$$L_{SUI}=A+10\gamma \log(\frac{d}{d_{0}})+S,d>d_{0}.$$ (3)

$$A=20\log\frac{4\pi d_{0}}{\lambda }.$$ (4)

$$\gamma =a-bh_{b}+\frac{C}{h_{b}}.$$ (5)

In which:

- *d* = distance from the antenna to the measured point, in meters.
- $d_{0}$ = 1 meter, reference distance according to [58].
- λ = wavelength, in meters.
- γ = path-loss exponent.
- $h_{b}$ = base station height, which can be between 10 to 80 meters.
- *a*, b and *c* = constants dependent on the terrain category, which can be seen in Table 1.
- *S* = shadowing effect, which can be between 8.2 and 10.6 dB.

**Table 1:** Type sizes and appearance.

Constant	Terrain A	Terrain B	Terrain C
**A**	4.6	4	3.6
**B**	0.0075	0.0065	0.005
**C**	12.6	17.1	20

By correctly assigning the input parameters, it is possible to simulate urban and suburban environments with shading [57].

Each user achieves Shannon´s capacity limit [59], i.e., the data rate for user *k* is expressed in (6) as:

$$CA_{k}=B\;\log2\left(1+SINR_{k}\right).$$ (6)

in which *B* is the bandwidth.

## Proposed heuristics solution procedure

The heuristics were divided into two groups with different spatial perspectives: one with a predefined location for the SCs and the other based on the users’ location. Two techniques were employed for the first group and one for the second. These are outlined below, together with their peculiarities, as well as their benefits and drawbacks, which will be shown in the results section.

### A. Heuristics based on pre-defined SC locations

Consider a geographical area A in which a number of SCs must be deployed. The candidate location model is given by *St = S *∪* Sf*, where *S* contains the possible places to install an SC without fiber optic connection and *Sf* locations with fiber backhaul connectivity. Note that these Fiber Access Points (FAPs) are also potential locations for the SC deployment since the SCs must be connected to the core network through some kind of backhaul solution. In the interests of simplicity, each *Sf* element is termed a node. The deployment variable $x_{i}$ can be defined as follows:

$$x_{i}=\{\begin{array}{c} 1,\;If\;the\;{\text{i}}^{\text{st}}\;node\;is\;selected\;to\;place\;a\;SC\\ 0,\;otherwise. \end{array}$$

Additionally, since the 5G networks access schemes have not yet been defined, the Orthogonal Frequency Division Multiple Access (OFDMA) was used as an alternative. OFDMA is based on the Orthogonal Frequency Division Multiplexing (OFDM) technique and thus acquires its immunity to InterSymbol Interference (ISI) in a frequency selective fading channel and offers good flexibility and a performance of reasonable complexity [60]. The users of the same cell are multiplexed in frequency, and the data of each user are transmitted on a subset of the sub-carriers of an OFDM symbol. Adaptive resource allocation and link adaptation techniques are essential to achieve the challenging targets of spectral efficiency and user throughput targets.

In OFDMA systems, resource allocation techniques can make use of the time and frequency variations of the system to optimize the use of the available resources. They exploit the available Channel State Information (CSI) at transmitter side so that they can carry out the power allocations and share the subcarriers with the users [60].

As stated in [32], it was assumed that *N* subcarriers were available for downlink transmission and that there was a predefined user distribution in an area of interest. A simple model was employed for the non-heterogeneous distribution of users, by randomly distributing them over the whole map (divided into quadrants). Four dense areas were created to characterize the office spaces, (this distribution will be illustrated in the results section).

The objective of the heuristics is to find the minimum number of SCs that can still ensure coverage and provide capacity requirements planning for all the users, while at the same time reducing the total cost of deployment, including that of both the wireless and wired infrastructure. It can be assumed that each user can only be served by exactly one cell and, thus, user demand is indivisible. In formal terms, the problem can be formulated as:

$$Min\;TotalCost=\sum_{i=1}^{S}x_{i}.C_{i}$$ (7)

$$C_{i}=\frac{C_{a}}{Users_{i}}+(d_{pi,qi}\left(C_{Trn}+C_{Fib}\right)*Z)$$ (8)

Where:

- $x_{i}$ = The Binary variable that assumes a value of 1 if the SC was deployed;
- $C_{a}$ = The fixed Cost of a SC deployment;
- $C_{i}$ = Total Cost of SC*i*;
- $C_{Trn}$ = Trenching cost per unit;
- $C_{Fib}$ = Fiber cost per unit;
- pi = Position where SC*i* was deployed;
- $q_{i}$ = Position of the point of access for SC*i*;
- $d_{p,q}$ = Distance between point *p* to *q* based on Taxicab geometry;
- ${Users}_{i}$ = Number of users that are assigned in the SC*i*;
- Z = Binary variable that assumes a value of 1 if it includes the Transport Costs in the optimization process;

$$d_{p,q}=\sum_{i=1}^{n}\left|p_{i}-q_{i}\right|$$ (9)

In addition to the objective function, there are some constraints that need to be noted:

$$\frac{\sum_{i\in I}\sum_{k\in K}A_{k,i}}{TotalUsers}\geq X$$ (10)

$$CA_{k,i}\geq CA_{min}$$ (11)

$$PRBs_{d,i}\leq PRBs_{t,i}$$ (12)

In Eq 10, $A_{k,i}$ = 1 means that user *k* was assigned to SC*i*, so this restriction guarantees a minimum percentage (value of *X*) of covered users. Eq 11 ensures that Shannon’s capacity (received by the user *k* from SC*i*) must have a minimum value and Eq 12 certifies that the number of PRBs delivered per SC*i* will not be greater than the total number of existing PRBs.

The heuristic Type 1 (T1) is only based on the SC cost (Z = 0) and the Type 2 (T2) on the total deployment cost (SC and Transport). In both cases all the candidate SCs are tested one at a time, and then the SC is removed, to find out if it was dispensable or indispensable.

Below are presented the proposed heuristics that aim to select the SCs necessary to cover a minimum coverage (coverage_min) and QoS (QoS_min) to the all users (UE).

**Algorithm 1 Network_Parameters_Check (Heuristics Type 1 and 2)**

**Input:** List of SCs (*St)*, *Z*, *UEs*

**Result:** *St* sorted by cost in descending order

1. **for all** *i* ∈ *St*

2.     Allocate UEs closest to *i*;

3. **end for**

4. **for all** *i* ∈ *St*

5.     Calculate the deployment cost of *i* (Using variable Z and Eqs 7, 8 and 9);

6.     *St* = St sorted by cost in **descending** order;

7. **end for**

8. **for all** *k* ∈ *UE*

9.     Update SINR of *k* according to Eq 1;

10.    Calculate Shannon capacity of *k* according to Eq 6;

11. **end for**

**Algorithm 2 Network deployment based on pre-defined SCs locations (Heuristics Type 1 and 2).**

**Input**: *St*, *Z*, *UEs*, *QoS_min*, *coverage_min*

**Result:** *St*

1. Network_Parameters_Check (*St*, *Z*, *UEs)*

2. **for all** *i* ∈ *St*

3.     *St’ = St;*

4.     *St* = remove *i* from *St*;

5.     Network_Parameters_Check (*St*, *Z*, *UEs*)

6.     **if** (UEs coverage ≥ *coverage_min*) && (UEs capacity ≥ *QoS_Min*)

7.         *St* = *St;*

8.     **else**

9.         *St = St’*;

10.     **end if**

11. **end for**

12. Network_Parameters_Check (*St*, *Z*, *UEs)*

13. Calculate the trenching using *Prim’s* Algorithm (*St*).

The initial of the algorithm 1 describes the process of allocating UEs to the closest SCs (where *St* is the list of possible SCs). After this phase, is calculated the deployment cost of each *i* ∈ *St* using Eqs 8, 9 and 10 (Z is used as a binary variable, to determine whether the cost of transport will be considered in the cost function), and then *St* is sorted by cost in descending order, *St* will serve as input to Algorithm 2. At the end of this phase, interference between SCs and UEs are calculated and the maximum capacity of each *k* ∈ *UE* is calculated, considering the resources available in each SCs (i.e., PRBs), which are divided evenly (regardless of the channel quality of the UE).

In Algorithm 2, in addition to the list (*St*), a minimum percentage of coverage (coverage_min) and throughput capacity (QoS_min) are given as input. In this algorithm, all *i* ∈ *S* is tested to determine if this SC is indispensable: the test is done by taking out SC and calculating whether the minimum requirements for coverage and QoS are met. If is, the SC is removed, otherwise, the SC *i* is deployed from *St*.

In the last phase, algorithm 1 is called again to recalculate the UEs assignment and Shannon capacity. After all tests, the remaining SC are deployed and are interconnected by means of Prim's algorithm [61], which is a greedy algorithm that finds a minimum spanning tree for a weighted undirected graph. This is a standard algorithm used worldwide in the literature for the purpose of making comparisons, although our framework is flexible enough to use any other algorithm.

### B. Heuristic based on the user´s location

Unlike the previous heuristics, the Type 3 (T3) does not include an initial set of SCs to be tested; instead, the users' positions are noted so that the semi-optimal locations to deploy SCs can be found. A K-means clustering algorithm was used to find these locations [62].

In the first stage, it is necessary to know the number of centroids (in this case, the number of SCs) that will be used. Before carrying out this task, it is essential to find out how many users each SC can serve. This number is obtained from the maximum capacity of an SC (in Physical Resource Blocks—PRBs) divided by the *PRBs_perUser* (number of PRBs, on average, that each user needs to meet the QoS requirements). The information on how many PRBs is available for each SC is provided by the *Total_PRBsPerSC* variable.

Algorithm 3 summarizes the heuristic. The variables (*PRBs_perUser* and *Total_PRBsPerSC*), the position of users (*UEs*) and the values of minimal coverage and QoS are received as input. The map is divided into several quadrants (all of the same size). The tasks of counting the number of users, detecting the number of SCs and calculating the position of the SCs (centroids), are carried out locally, in each quadrant. The algorithm checks all the users (one at a time) so that it can allocate them to a base station, a task that can only be undertaken if the SC has the number of PRBs required to meet the users’ predetermined requirements.

In line 12 is checked if the SCs created attend the minimal coverage and QoS of all users. In positive case, the Prim’s algorithm is called to calculate the trenching costs, and with this information is possible to know the total cost of deployment of all SCs. In negative case, the algorithm 3 is called recursively, an input value is increased (*PRBs_perUser*) and a new SC deployment scheme is formed, with more SCs. In this way, the capacity of the network is increased. This process is repeated until the values of *QoS_min* and *coverage_min* are satisfied.

**Algorithm 3 SCs deployment based on users’ locations (Heuristic Type 3).**

**Input**: *PRBs_perUser*, *Total_PRBsPerSC*, *UEs*, *QoS_min*, *coverage_min*

**Result:** *St*

1. *Q* = Divide the map into quadrants

2. **for all** *q* ∈ *Q*

3.     Count the number of UEs per quadrant

4.     Calculate the number SCs per quadrant (UEs x *PRBs_perUser/Total_PRBsPerSC)*

5.     *St* = Calculate the position of SCs (using K-means)

6. **end for**

7. **for all** *k* ∈ *UE*

8.     Pre-Assign each *k* to three-best-SINR SCs;

9.     Calculate the number of PRBs that each *k* needs to meet QoS requirements;

10.     Assign *k* to the SC with PRBs available;

11. **end for**

12. **if(UEs** coverage ≥ *coverage_min*) && (UEs capacity ≥ *QoS_Min*)

13.     Calculate the trenching using *Prim’s* Algorithm (*Sn*).

14. **else**

15.     Algorithm 3 ((*PRBs_perUser*+0.2), *Total_PRBsPerSC*, *UEs*, *QoS_min*, *coverage_min*)

16. **end if**

## Results

An example is given of an application of heuristics in a typical urban area in Stockholm (Sweden). The scenario under examination was modeled as a Manhattan street grid, and no macro layer was included.

**Fig 1:**
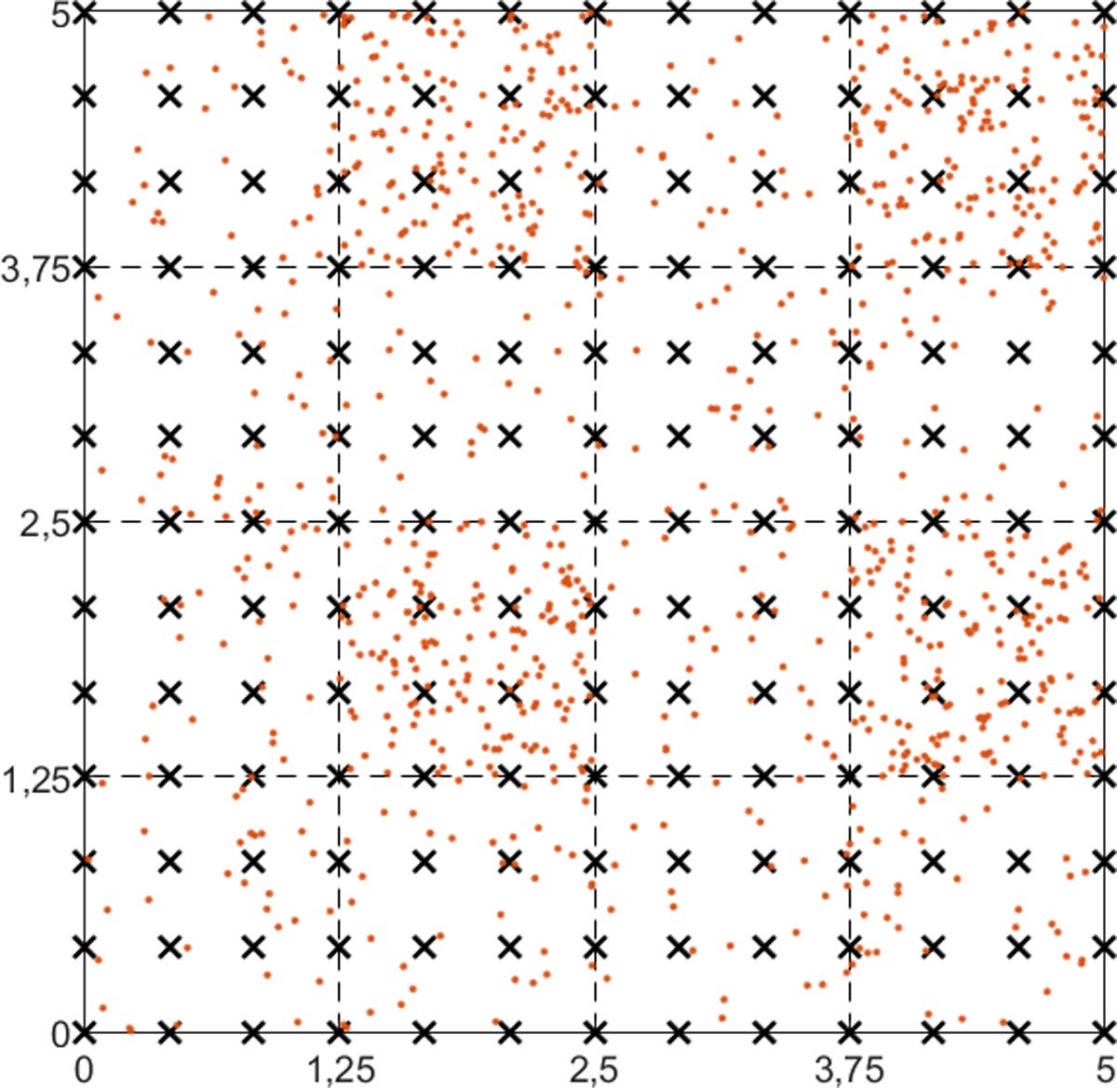
Example of an SC deployment with users divided into two geo-types (red dots); the black “x” represents the possible deployment of an SC.

As discussed in the earlier sections, areas with dense SC deployments were added to our simulated network to illustrate some of the key operations of the 5G networks. Fig 1 shows an example where a number of users are spread over the area. The top left and bottom right districts represent sparse residential areas (i.e., low concentration of users), the top right and bottom left districts are business areas (i.e., with a high concentration of features such as shopping malls, football stadiums, and locations with a high level of indoor traffic).

**Fig 2:**
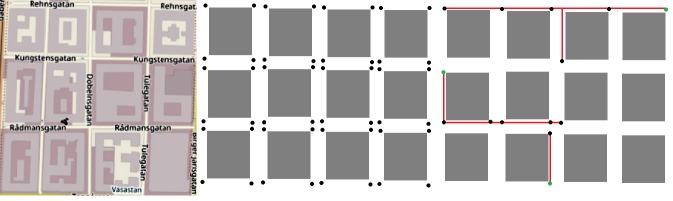
Example of the SC deployment process; the green squares represent the fiber access points.

The general simulation parameters are shown in Table 2 and Figs 1 and 2.

**Table 2:** Network Parameters.

Parameters	Value
Carrier frequency [GHz]	LTE-A 3.5 GHz
System bandwidth	20 Mhz, 100 PRBs per SC
Max. power [dBm]	20
Fiber cost ($C_{Fib}$)[29]	1
trenching cost ($C_{Trn}$) [29]	4 x Fiber cost
Small cell cost ($C_{a}$) [29]	375 x Fiber cost
Block sizes	416m x 416m
Local area map	5Km x 5Km
Minimal coverage	100%
Minimal QoS	400 Kbps

Road intersections were chosen as the intended locations for the SCs deployments (for the T1 and T2 heuristics). 169 possible nodes were selected for SC deployments in this scenario, and these are shown in the X dots (Fig 1). The FAPs were positioned in different places. The purpose of applying the heuristics (T1 and T2), is to identify the ideal locations for SC from the network of roads, and find the optimal routes for fiber planning that branch out from the existing fiber access points to these selected SC locations.

Fig 1 shows the divisions in the quadrant that were used in the T3 heuristic. Each quadrant is a block with a side that is 1.25Km long. Four of the sixteen quadrants have a large crowd of users, making up 12.5% of the total number; the other 50% are randomly distributed over the map, which means that these crowded areas may contain more than 12.5% of the users. These quadrants are the same as those used in the T3 heuristic to find the positions of the SCs.

The results were obtained after thirty iterations, in each of which the position of the users and FAPs were different. Another key factor is ¨user densification¨, which allowed scenarios to be created with four different numbers of users: 400, 600, 800, 1000.

The approaches were compared by means of the following performance measurements: the number of deployed SCs, total cost of deployment, resource distribution (Jain’s Fairness Index) and scalability. The measurement used to calculate the resource distribution was the Shannon capacity formula described in 6.

### A. Number of SCs deployed

**Fig 3:**
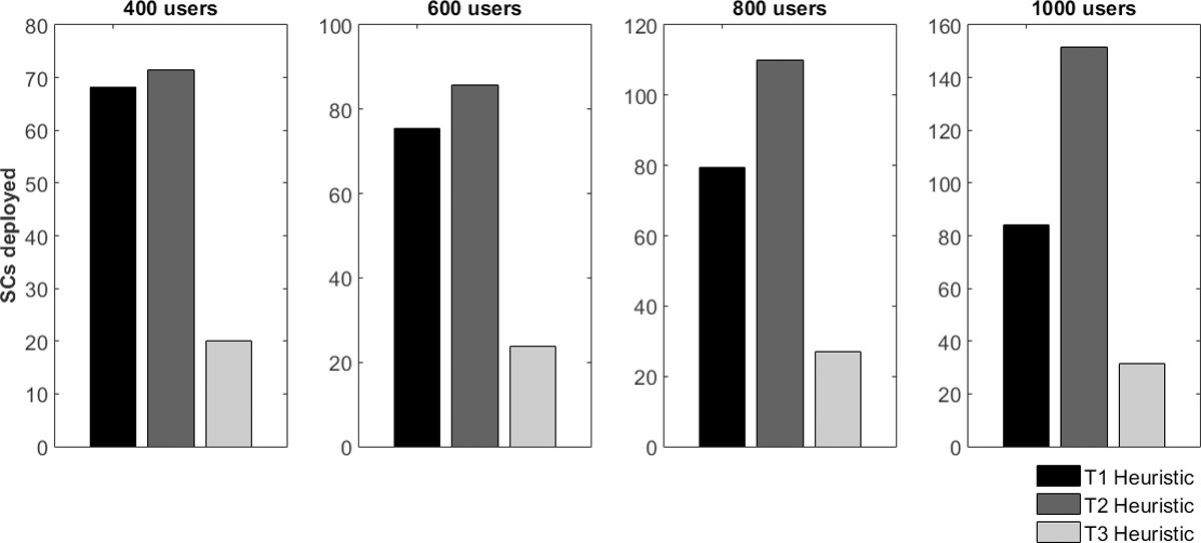
SCs deployed for the heuristics.

The results given in this section are linked to the number of SCs that each heuristic needed for coverage and to meet the QoS requirements of all the users. Fig 3 shows the (average) number of SCs deployed.

The heuristic T1 roughly needed between 68 SCs (400 users) and 84 SCs (1000 users) to properly serve the network, which led to a reduction of approximately 60% of SCs (400 users) and 50% (1000 users). This approach is based on the concentration of users in the same SC. The T2 heuristic had a lower performance; and only achieved a reduction of 58% (400 users) and 11% (1000 users). This can be attributed to the choice of SCs, as well as the positioning of the FAPs and, hence, the trenching required to meet this backhaul requirements, which are traditionally the most expensive, even if the number of SCs is higher.

The T3 heuristic achieved better results and between 20 and 32 SCs were needed to serve all the users. The avoidance of street networks orientations and pre-defined positions dramatically affected the results. It should be noted that this approach depends a great deal on free areas, which limits its scope.

### B. Total cost of deployment

**Fig 4:**
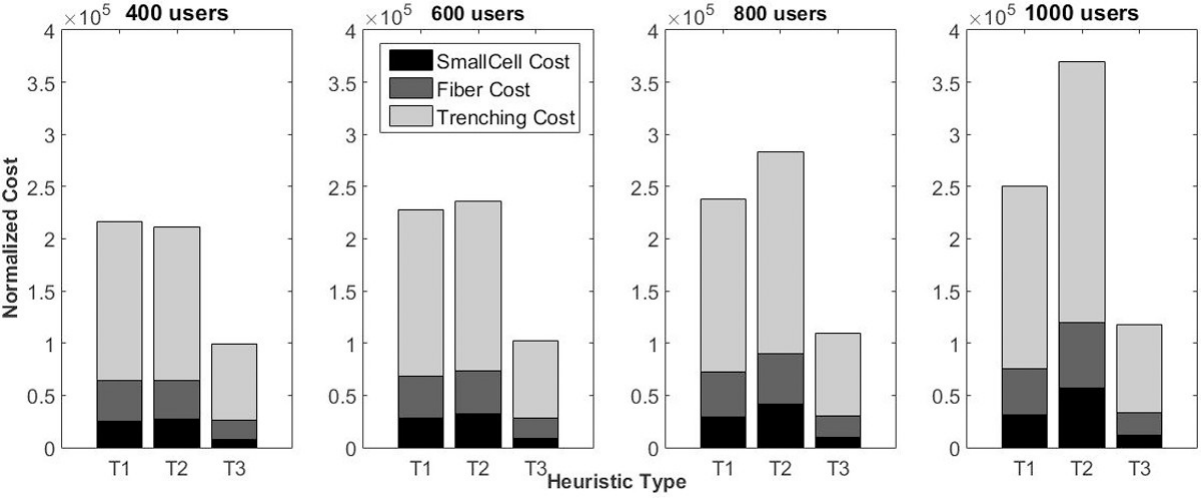
Normalized deployment cost of the three heuristics with different degrees of user densification.

Fig 4 shows the total cost incurred by the T1, T2 and T3 heuristics, calculated by Eqs 8 and 9, no matter which heuristic was used. On the basis of the results (Fig 4), it is clear that the T2 heuristic performs better in scenarios with low densification and approximate numbers of SCs that need to be installed,. In other cases (when there are 600, 800 and 1000 users), the difference in the number of points to be covered, raises the total cost of SCs, fiber (per km) and, hence trenching (per km), which makes the T1 heuristic more efficient. These results demonstrate that the search for optimized scenarios using RNP and transport, at the same time, may not lead to satisfactory results.

As expected the T3 Heuristic, incurred the lowest costs. Compared with T1, there is a reduction of approximately 50% of the total cost, rising to 55% in dense scenarios. More significant results were found when a comparison was made between T3 and T2: a reduction of 50% with 400 users with an increase in later scenarios, reaching 76% in results with 1000 users.

**Fig 5:**
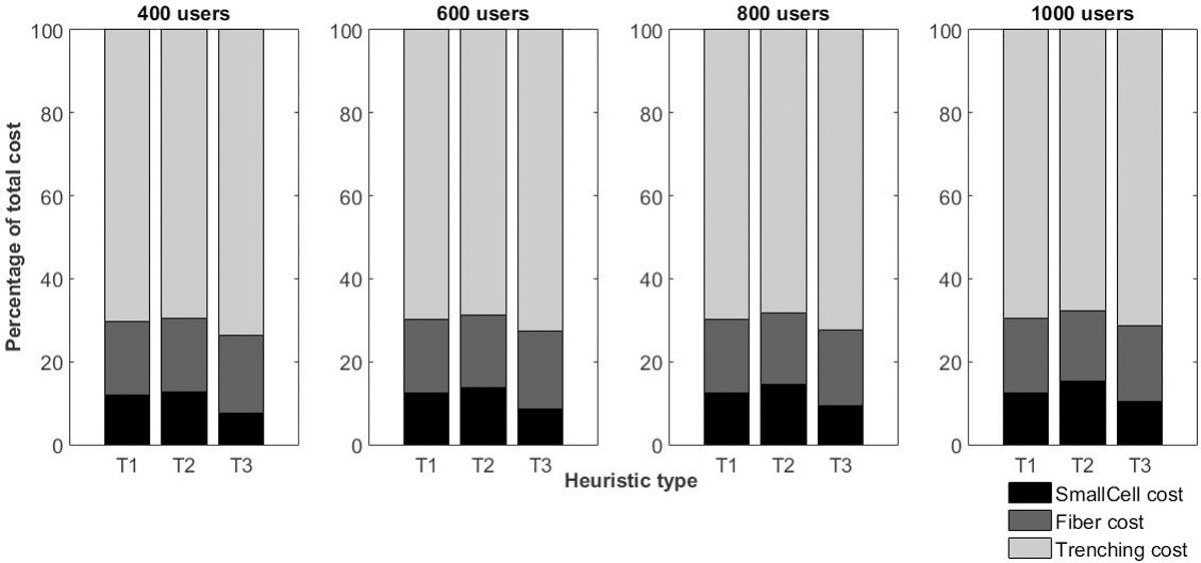
Percentage of total cost of the resources used.

In the deployment of large networks, it is essential to know which resource will need more investment. With regard to this, Fig 5 shows the percentage of each item in terms of the total cost.

As stated in [29], the trenching cost represents a significant proportion of the total cost. On average (for all densification scenarios), the total cost of the TI heuristic consists of the trenching cost (69.6%), fiber cost (17.8%) and SC deployment cost (12.6%); in T2, 67.6% is made up of trenching, 17%, fiber and 15.4%, SCs; finally, in T3, the trenching cost is responsible for 71.4% of the total cost. The rest is divided between fiber (18.2%) and SCs (10.4%).

Although these values are very close in percentage terms (e.g., in a scenario of 1000 users, the trenching costs is responsible for 69.6% of the total cost in T1 and 71.4% in T3), the absolute values are very different (in T1 they are U$173,333.00 and in T1 U$ 83,368.00 of normalized values), as can be observed in Figs 4 and 5. The same applies to the other parts of the total cost.

### C. Resource distribution and scalability

The main purpose of the algorithms is to attend the users and meet the QoS requirements;. Jain’s Fairness Index is a coefficient that is used to determine if users are being allocated a fair share of the resources (in this case, evaluate if the resources are attending the minimum QoS and are well distributed among the users).

In LTE mobile networks, the resources are represented by the PRBs and are affected by distance and signal path loss conditions [63]. Each user may need some or even dozens of PRBs to achieve the established minimum capacity. In view of this, Jain’s Fairness Index was calculated by means of the Shannon capacity of each user.

Table 3 shows the results obtained from Jain’s Fairness Index. The T3 approach obtained the best results and a minimal variation between the users’ densification. This means that the algorithm was able to maintain the quality of this metric. On the other hand, the T1 and T2 techniques had wider variations although they were not so significant. It should be noted that, in numerical terms, the T3 heuristic distributed the network resources approximately 2.5 times better than the other two.

**Table 3:** Jain’s fairness index.

Number of users	T1	T2	T3
400	0.259	0.279	0.684
600	0.240	0.285	0.684
800	0.238	0.297	0.687
1000	0.249	0.278	0.685

The fact that the distribution of the resources in T3 was better than T1 and T2 can be attributed to the optimized allocation of the SCs, which allow a better distribution of the users. However, if there are significant changes in the number of users (the tidal effect), it means the T3 approach has already reached its limit, while the other approaches have resources that can be redistributed. The T2 approach in particular, obtained a larger number of installed BSs and, thus, was able to have more PRBs for allocation.

This factor is illustrated by Table 4 and proved by previous results where there was a considerable growth in the number of the SCs deployed. As a result, there was a large number of PRBs (especially in crowded areas), and this created the most scalable network.

**Table 4:** Shannon capacity (Gbps).

Number of users	T1	T2	T3
400	4.682	5.172	0.325
600	4.893	6.252	0.521
800	5.141	7.012	0.721
1000	5.579	13.002	0.904

## Conclusion

SCs provide an increase in coverage and capacity by offloading macrocells and thus are able to alleviate the congestion of the mobile network, which was not initially designed for data traffic. The optimal allocation of SCs is still an open problem and becomes even more complex when factors such as the transport network and minimal QoS are taken into account. By adopting a heuristic approach for their allocation, this study has sought to provide users with the minimum levels of quality of QoS and reduce the total cost of SC and transport deployment. The results showed a significant reduction in the total cost and to a great extent, relied on the user distribution and position of the FAPs.

The benefits resulting from this study are threefold: (a) proposal of a new heuristic for SC deployment based on a clustering approach; (b) unlike other studies in the literature, the heuristic was formed by including cross-layer factors (throughput and SINR); (c) it allows a comparison to be made between different approaches based on SC and the transport deployment heuristic for next generation mobile networks.

It should be stressed that this work does not consider the concept of Complex networks. This has attracted a great deal of attention among researchers and involves many important phenomena which cannot be encompassed by isolated networks. Since real-world complex systems are becoming increasingly dependent on each other, the study of interdependent networks has become a key issue in network science.

The investigation into complex real-world systems (such as the relation between mobile access networks and power systems, for example) has shown that interdependent networks can lead to new discoveries that cannot be explained by establishing a single-network framework [60]. Problems like vulnerability to attacks, dynamics and synchronization behavior scale-free onion like networks or the structural controllability of a network within complex networks, are not addressed here. However, in future work, we intend to study further the optimization problem that arises from deploying radio networks, while taking account of these factors.

Finally, with regard to the limitations of this study, the following areas should be mentioned here as subjects of future work: the changing position of users over a given period of time; the different radius for SCs and an evaluation of the traffic requirements for different applications.
